# Coronary stent-graft use to salvage a juxta-anastomotic arterial rupture complicating a case of radio-cephalic fistuloplasty

**DOI:** 10.1186/s42155-022-00323-0

**Published:** 2022-08-20

**Authors:** Zhiyuan Lin, Neha Kallam, Ruhaid Khurram, Ammar Al Midani, Mohamed Khalifa

**Affiliations:** Royal Free Hospital, Pond St, London, NW3 2QG UK

**Keywords:** AVF stenosis, Fistuloplasty, Vessel rupture, Pseudoaneurysm, BeGraft coronary stent-graft

## Abstract

**Background:**

Stenosis is a common complication of haemodialysis arteriovenous accesses. Endovascular approaches with percutaneous transluminal fistuloplasty have largely replaced open surgical approaches as first line treatment. Vessel rupture is an uncommon complication of fistuloplasty and most reports describe venous rupture. Stent-graft deployment can salvage this, however, its use requires careful assessment of the distal vasculature. Arterial rupture with fistuloplasty has rarely been described in the literature. This is a novel case describing the use of a BeGraft coronary stent-graft to manage juxta-anastomotic arterial rupture and pseudoaneurysm complicating fistuloplasty.

**Case presentation:**

A 77 year old female with end stage renal failure secondary to systemic amyloid light chain type amyloidosis was referred for a suspected radio-cephalic arteriovenous fistula stenosis after difficulty cannulating with poor flow during dialysis and clinical reduction in the fistula thrill. Both Doppler ultrasound and intravenous fistulography confirmed a venous stenosis 2 cm distal to the anastomosis. The stenosis was treated by fistuloplasty, however, this was complicated by a rupture of the juxta-anastomotic arterial segment intraoperatively. Intermittent balloon tamponade was used to minimise extravasation although a pseudoaneurysm formed within the damaged arterial segment. The patient’s distal neurovascular status was assessed using the Barbeau test and we sonographically confirmed adequate retrograde arterial flow via a complete palmar arch directing blood from the ulnar artery. After discussion with the renal transplant team, a 4 mm BeGraft coronary stent-graft was deployed to control haemorrhage and bypass the pseudoaneurysm until adequate haemostasis and fistula flow was achieved. Follow-up 3 months post-procedure reported the patient continued with haemodialysis using the stented fistula with no further complications.

**Conclusions:**

To our knowledge, this is the first case report describing the application of BeGraft coronary stent-grafts to salvage fistuloplasty of a radio-cephalic arteriovenous fistula stenosis complicated by juxta-anastomotic arterial rupture and pseudoaneurysm formation. We demonstrate the safety and short-term efficacy of this technology.

## Background

Fistuloplasty, or percutaneous transluminal angioplasty (PTA), remains the first-line treatment of arteriovenous fistula (AVF) stenosis with a technical success rate of 90% (Beathard et al. 2004). There is a small but significant complication rate for fistuloplasty of approximately 4%, with haematoma formation being the most common complication (Beathard et al. 2004). Vessel rupture occurs in 0.9%–3.8% of fistuloplasties in the management of haemodialysis arteriovenous (AV) access stenosis (Raynaud et al. 1998; Kornfield et al. 2009; Bittl 2009). However, most reports of iatrogenic vessel rupture are venous.

Vessel rupture can be managed with balloon tamponade, stent-graft deployment or conservatively with external compression and intentional thrombosis. Several studies have demonstrated the technical success, long-term efficacy and safety of stent-grafts for failed or complicated fistuloplasty. Across these studies, the technical and clinical success rate was between 98%–100% (Bent et al. 2010; Dolmatch et al. 2012; Özkan et al. 2013; Schmelter et al. 2014).

We report the case of a 77 year old woman who underwent fistuloplasty for a radio-cephalic AVF with inflow stenosis which was complicated by rupture of the juxta-anastomotic arterial segment and pseudoaneurysm formation requiring stent-graft deployment.

## Case report

A 77 year old female with end stage renal failure secondary to systemic amyloid light chain (AL) type amyloidosis on long term haemodialysis was referred to the interventional radiology department following difficulty in cannulating her AVF and clinical reduction in the palpable fistula thrill. Her significant medical comorbidities include type 2 diabetes mellitus, hypertension, hypercholesterolaemia and a pacemaker for Mobitz type 2 second degree atrioventricular block.

Doppler ultrasound revealed a stenosis 2 cm distal to the anastomosis. With systemic heparinisation, we performed fistulography under intravenous sedation (2 mg Midazolam, 50 μg Fentanyl) and local anaesthetic (10 ml 1% Lidocaine). Glyceryl trinitrate (800 μg) and Amlodipine (10 mg) were administered during the procedure due to a systolic blood pressure of over 200 mmHg on a background of known hypertension. This controlled the systolic pressure to 150 mmHg.

**Fig. 1:**
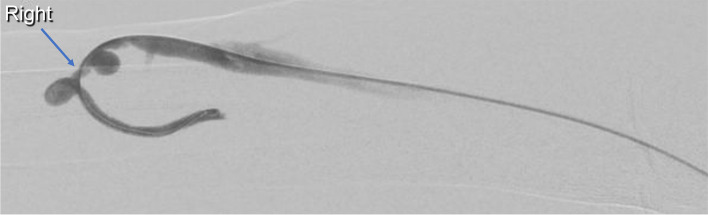
An initial fistulogram demonstrating venous puncture and successful passage of the catheter into the radial artery with a juxta-anastomotic venous segment stenosis (blue arrow)

Under ultrasound guidance, retrograde puncture of the fistula was performed. A 5F sheath was inserted and the catheter and wire were successfully passed across the stenotic lesion. The initial venogram confirmed a single juxta-anastomotic venous segment stenosis (Fig. 1).

**Fig. 2:**
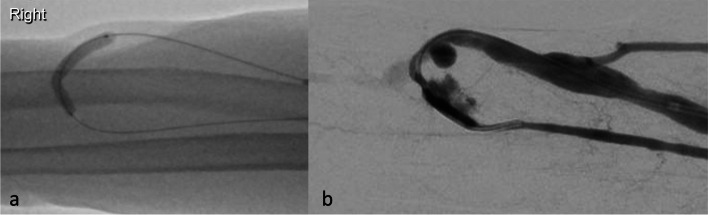
Short segment of the Sterling balloon inflated within the juxta-anastomotic arterial segment in an attempt to achieve optimal dilation (**a**). Active extravasation of contrast secondary to a rupture of the juxta-anastomotic arterial segment (**b**)

This was initially dilated with a 4 mm Sterling balloon (Boston Scientific) which resulted in suboptimal reduction of the stenosis after which a 5 mm Sterling balloon was used to overcome the tight stenosis. To achieve optimal dilatation of the affected vessel, a short segment of the balloon was positioned and inflated within the arterial segment to attain adequate vessel patency and to prevent migration of the balloon proximally due to arterial-flow back pressure. This was complicated by rupture at the juxta-anastomotic radial artery segment which immediately presented as pain and swelling in the patient’s forearm and was confirmed by fistulography (Fig. 2).

Intermittent balloon tamponade was maintained with a 3 mm balloon at the level of contrast extravasation. To ensure safety of the distal limb whilst the balloon was inflated, the neurovascular status of the patient was clinically assessed together with ultrasound of the radial artery distal to the fistula which demonstrated adequate retrograde blood flow from the complete palmar arch via the ulnar artery. This was further confirmed with a Barbeau test which showed no dampening of the pulse oximeter waveform on the ipsilateral thumb with balloon inflation. Confirmation of the above allowed for prolonged balloon tamponade for a total duration of 30 minutes without concern for neurovascular compromise to the distal limb.

**Fig. 3:**
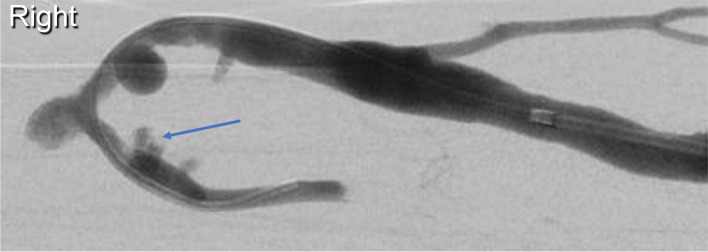
Formation of a pseudoaneurysm (blue arrow) within the short arterial segment

**Fig. 4:**
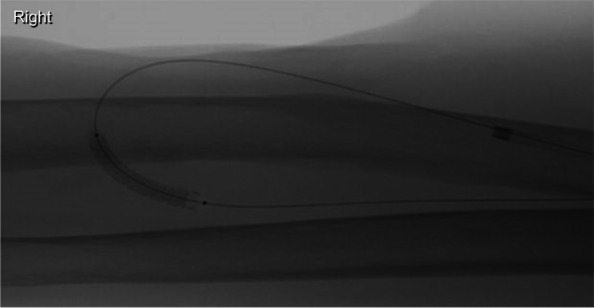
4 mm BeGraft coronary stent-graft deployed within the arterial portion of the anastomosis to bypass the pseudoaneurysm

Whilst intermittent balloon tamponade minimised extravasation, a small pseudoaneurysm subsequently formed within the short arterial segment (Fig. 3). After discussion with the renal transplant team, a joint decision was made to deploy a covered stent-graft within the arterial portion of the anastomosis. A 4 mm BeGraft coronary stent-graft (Bentley InnoMed GmbH) was deployed in an adequate position within the arterial portion of the anastomosis (Fig. 4).

**Fig. 5:**
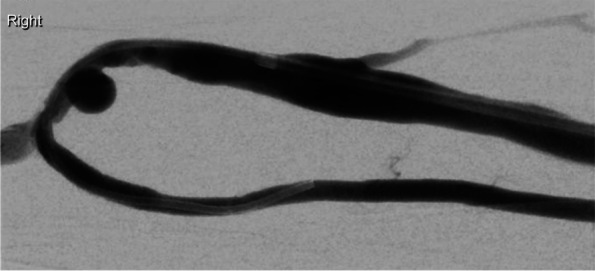
Completion fistulogram demonstrating no evidence of active contrast extravasation following successful stent-graft placement into the arterial segment

The completion fistulogram demonstrated excellent flow through the fistula with no evidence of active bleeding and complete exclusion of the pseudoaneurysm (Fig. 5). There was no clinical evidence of compartment syndrome.

The patient recovered well post-procedure and was admitted overnight for monitoring and neurovascular observations. Her post-operative haemoglobin levels were within normal limits (113 g/L; ref.: 110-150 g/L). The forearm swelling improved significantly, and no neurovascular complications were noted the following morning. The patient was reviewed in haemodialysis clinic 3 months later and successfully continued with haemodialysis through the stented fistula, reporting no further complications.

## Discussion

PTA induced arterial rupture is a rare but serious complication in percutaneous coronary intervention and has also been reported for femoral, popliteal, iliac and renal artery interventions (Yeo et al. 2008). However in AVFs, whilst venous rupture is well-documented, there is a paucity of literature on arterial rupture.

As per standard practice, a retrograde transvenous puncture was used. Whilst some studies have discussed the putative advantages of an arterial approach in treating multiple downstream lesions with a single sheath, we considered the retrograde transvenous approach as the most appropriate in our scenario as only a single juxta-anastomotic lesion had been identified and good access and control had been established from the initial puncture site (Le et al. 2015). This also avoided potential complications of arterial puncture including arterial occlusion, vasospasm, further rupture and difficulties with closure and haemostasis.

There are few specifically designed or licensed stents for use in AVFs and a wide variety of stent types have been tried off-label. To the best of the authors’ knowledge, this is the first case report describing the use of a BeGraft coronary stent-graft, a balloon-expandable expanded-polytetrafluoroethylene (ePTFE) covered Cobalt-Chromium stent-graft, to manage juxta-anastomotic arterial rupture and pseudoaneurysm complicating fistuloplasty for a radio-cephalic AVF stenosis.

Whilst BeGraft is commonly used in coronary intervention, a small number of studies have reported application of a BeGraft coronary stent-graft within a comparable non-coronary context such as in traumatic or iatrogenic injury to the anterior tibial artery and other small vessels including the renal, gluteal and ascending cervical arteries (Brunoro et al. 2019; Ruffino et al. 2020).

Due to the small calibre of the radial artery and the short segment requiring exclusion, a low-profile covered stent was required to manage this. Stent-grafts traditionally used for peripheral interventions such as the Viabahn (WL Gore & Associates) or Fluency Plus (Bard), both self-expanding ePTFE covered Nitinol stent-grafts, would not have met these requirements due to their larger profiles. Furthermore, balloon-expandable stent-grafts allow for more precise positioning as they do not require stepwise deployment, making the BeGraft coronary stent-graft the most suitable option for this application.

Sufficient segments of proximal and distal landing zones were identified for stent-graft placement to adequately exclude the pseudoaneurysm without crossing the anastomosis. Had an adequate distal arterial landing zone not been present, it would have been feasible to cross the anastomosis with the stent-graft as we had demonstrated adequate retrograde flow from the ulnar artery via the intact palmar arch. This alternative placement would have been suboptimal as the angulation of the anastomosis would have increased the risk of stent fatigue. Both explant and in silico data demonstrate that pulsatile fatigue-related damage of stent-grafts concentrate in areas of severe angulation and bending (Dalbosco et al. 2020; Zarins et al. 2004).

Irrespective of the approach used to treat AVF arterial rupture and pseudoaneurysm, whether balloon tamponade or stent-graft deployment is used or the positioning of the stent-graft, it is vital to assess for adequate retrograde blood flow via the palmar arch from the ulnar artery. This can be clinically proven by neurovascular assessment, the Barbeau test and sonographically by ultrasound of the distal vasculature. If necessary, prolonged tamponade in arterial rupture and stent-grafting across the anastomosis can be safe with confirmation of the above.

Despite the high technical success rate demonstrated by stent-grafts in both AVF vessel rupture and pseudoaneurysms, there are a few limitations. Covered stent-grafts are not designed for repeat cannulation which means once deployed, the area must be marked to avoid cannulation over that site as in our patient.

## Conclusion

In conclusion, juxta-anastomotic arterial segment rupture is a rare complication of fistuloplasty that can be managed with endovascular stent-graft deployment. Clinical assessment of the neurovascular status and adequate retrograde palmar arch flow via the ulnar artery are key to ensuring safe balloon tamponade and stent-graft deployment. We demonstrate that the BeGraft coronary stent-graft is safe and effective in the short-term. Its long-term success in salvaging failed or complicated fistuloplasty of AV accesses requires further investigation.

## Data Availability

Not applicable.

## Abbreviations

AV: Arteriovenous
AVF: Arteriovenous fistula
ePTFE: Expanded polytetrafluoroethylene
PTA: Percutaneous transluminal angioplasty
